# A Diet High in Saturated Fat and Sucrose Alters Glucoregulation and Induces Aortic Fatty Streaks in New Zealand White Rabbits

**DOI:** 10.1080/15604280214274

**Published:** 2002

**Authors:** Weidong Yin, Zhonghua Yuan, Zongbao Wang, Baotang Yang, Yongzong Yang

**Affiliations:** 1 Institute of Cardiovascular Research Nanhua University Medical College Hengyang, Hunan 421001 China; 2 Department of Pathophysiology Central South University Xiangya Medical College Changsha, Hunan China

## Abstract

A new and convenient animal model for studying peripheral vascular and coronary artery disease in diabetes was established in this study. Male New Zealand White rabbits weighing approximately 2 kg were divided into 2 groups: a normal control group fed standard laboratory chow and a diabetogenic diet–fed group received a high-fat/high-sucrose diet. The high-fat/high-sucrose diet (contained 10% lard and 37% sucrose) feeding was maintained for 6 months. Plasma total cholesterol, high-density lipoprotein (HDL) cholesterol, triglyceride, superoxide dismutase, nitric oxide, nitric oxide synthase, insulin, and glucose were quantitated at monthly or bimonthly intervals. The aortic fatty streak lesions were quantified following lipid staining with Sudan IV. The aortic samples were observed by electron microscopy. High plasma triglyceride and glucose concentrations were induced. At the end of 6 months, the aortic fatty streak lesions were present in the animals' vascular specimens. As far as we know, this is the first report that demonstrates that New Zealand White rabbits can develop obvious aortic fatty streaks by feeding a high-fat/high-sucrose diet. Our results suggest that NewZealand White rabbits fed a high-fat/high-sucrose diet would provide a convenient model for studying peripheral vascular and coronary artery disease in diabetes.


*Int. Jnl. Experimental Diab. Res.*, 3:179–184, 2002
Copyright © 2002 Taylor & Francis
1560–4284/02 $12.00 + .00
DOI: 10.1080/15604280290013883

Taylor & Francis healthsciences

### A Diet High in Saturated Fat and Sucrose Alters Glucoregulation and Induces Aortic Fatty Streaks in New Zealand White Rabbits

**Weidong Yin,[1,2] Zhonghua Yuan,[1,2] Zongbao Wang,[1] Baotang Yang,[1] and Yongzong Yang[1]**

[1] *Institute of Cardiovascular Research, Nanhua University Medical College, Hengyang, Hunan, People's Republic of China*
[2] *Department of Pathophysiology, Central South University Xiangya Medical College, Changsha, Hunan, People's Republic of China*

**A new and convenient animal model for studying peripheral vascular and coronary artery disease in diabetes was established in this study. Male New Zealand White rabbits weighing approximately 2 kg were divided into 2 groups: a normal control group fed standard laboratory chow and a diabetogenic diet–fed group received a high-fat/high-sucrose diet. The high-fat/high-sucrose diet (contained 10% lard and 37% sucrose) feeding was maintained for 6 months. Plasma total cholesterol, high-density lipoprotein (HDL) cholesterol, triglyceride, superoxide dismutase, nitric oxide, nitric oxide synthase, insulin, and glucose were quantitated at monthly or bimonthly intervals. The aortic fatty streak lesions were quantified following lipid staining with Sudan IV. The aortic samples were observed by electron microscopy. High plasma triglyceride and glucose concentrations were induced. At the end of 6 months, the aortic fatty streak lesions were present in the animals' vascular specimens. As far as we know, this is the first report that demonstrates that New Zealand White rabbits can develop obvious aortic fatty streaks by feeding a high-fat/high-sucrose diet. Our results suggest that New Zealand White rabbits fed a high-fat/high-sucrose diet would provide a convenient model for studying peripheral vascular and coronary artery disease in diabetes.**

**Keywords** Atherosclerosis; Diabetes; Diabetogenic Diet; New Zealand White Rabbits

Type 2 diabetes (non–insulin-dependent diabetes mellitus, NIDDM) is a major risk factor for atherosclerosis. At equivalent conventional risk levels, there is a 4- to 5-fold increase in the mortality from vascular disease in diabetic patients, for example, coronary heart disease caused by atherosclerosis [1, 2]. There is also evidence that elevated triglycerides is an important cardiovascular risk factor, especially in diabetics [3]. The mechanism by which diabetes accelerates the development of atherosclerosis needs to be further elucidated. Satisfactory animal models for studying the relationship between components of diabetes, such as insulin resistance, dyslipidemia, and the occurrence and progression of atherosclerosis, are currently sparse [4–6].

The cholesterol-fed rabbit has been a widely used model for experimental atherosclerosis research [7]. This model can be combined with a number of other methods causing endothelial dysfunction, diabetes, artificial hypertension, or infection [7]. Nevertheless, to our knowledge, the rabbit has not previously been used to induce hyperglycemia by a diet high in saturated fat and glucose for the study of diabetes associated atherosclerosis.

In this study, New Zealand White rabbits were fed a high-fat/high-sucrose diet for up to 6 months, and the plasma parameters and the development of aortic fatty streak lesions were investigated. Ultrastructural pathological changes were also studied. We demonstrate that this diet induced an altered plasma lipoprotein profile, hyperglycemia, and obvious aortic fatty streak lesions in the rabbits.

Received 4 March 2002; accepted 23 May 2002.
This study was supported by a fund for returned overseas scholars from the Chinese Ministry of Education to Weidong Yin.
Address correspondence to Weidong Yin, PhD, Professor, Institute of Cardiovascular Research, Nanhua University Medical College, Hengyang, Hunan 421001, People's Republic of China. E-mail: wdy20012001@yahoo.com

### MATERIAL AND METHODS

### Animals and Diets

Male New Zealand White rabbits, weighing approximately 2 kg, were divided into 2 groups, a normal control group (C) fed standard laboratory chow (n = 12), and a diabetogenic diet–fed group (D) (n = 12) received a high-fat/high-sucrose diet (prepared in our institute, contained 10% pork lard and 37% sucrose). The complete composition of the above diets was given in Table 1. Animals were maintained in a temperature-controlled (22°C) facility with a 12-hour light/dark cycle, given free access to food and water, and acclimatized for 2 weeks before the start of the experiment. Body weight was recorded on a monthly basis. Blood samples were withdrawn from auricular veins at the baseline and at the end of each month after an overnight fast. At the end of the experimental period, the animals were sacrificed by phlebotomy under anesthesia with sodium pentobarbital, and the aortas were perfused and collected. The whole experimental period was 6.5 months.

All animal experiments were approved by the local animal ethics committee of Nanhua University Medical College.

### Plasma Assays

Plasma total cholesterol (TC), high-density lipoprotein cholesterol (HDLc), triglyceride (TG), superoxide dismutase (SOD), nitric oxide (NO), nitric oxide synthase (NOS), and glucose were quantitated using commercially available kits (Rongshen Biotech, Shanghai, China). Plasma insulin was measured using a radioimmunoassay kit (Academy of Atomic Energy, Beijing, China). Plasma peroxides were measured as thiobarbituric acid reactive substances (TBARSs) and expressed as equivalent nmoles of malondialdehyde (MDA). SOD, NO, NOS, and MDA were measured at bimonthly intervals; others were measured at monthly intervals.

**TABLE 1**
The composition of the two diets

| Composition | Regular chow (%) | High-fat high-sucrose diet (%) |
|---|---|---|
| Corn | 15 | 8 |
| Wheat | 10 | 5 |
| Rice | 10 | 5 |
| Grass powder | 30 | 15 |
| Bran | 30 | 15 |
| Fish powder | 2 | 1 |
| Bone powder | 0.5 | 0.5 |
| Soybean | 2 | 1 |
| Salt | 0.5 | 0.5 |
| Trace element | little | little |
| Vitamins | little | little |
| Pork lard | | 10 |
| Sucrose | | 37 |
| Cholesterol (nutrients) | | 9.3 mg |
| Carbohydrates | 53.2 | 65.09 |
| Protein | 11.1 | 5.88 |
| Fat | 2.42 | 11.28 |

### Aortic Lesion Analysis

The aortas were removed from the animals, cleaned of peripheral fat under a dissecting microscope, opened longitudinally, and the fatty streak lesions were quantified by a dot-counting method following lipid staining with Sudan IV. Templates of the vessels were drawn on clear acrylic sheets and superimposed over a dot grid with a 2 × 2-mm grid size. The number of dots in lesioned areas and in whole aortic area was counted.

### Ultrastructural Study

For transmission electron microscopy (TEM), perfused aortic samples selected from the arch and the thoracic aorta were postfixed in 1% $O_SO_4$ for 2 hours and dehydrated through an alcohol series and propylene oxide before they were embedded in Epon 812. Ultrathin sections were cut by an ultramicrotome, counterstained with uranyl acetate and lead citrate, and studied under a Philips 301 electron microscope.

For scanning electron microscopy (SEM), arterial fragments were dehydrated in ethanol and acetone series and dried in an E3100 critical point drier with $CO_2$ transition fluid. Specimens were mounted on aluminum stubs with silver print and coated with a 20-nm gold layer in an E500-PS3 sputter-coater. Photographs were taken using a SCAN100 scanning electron microscope at 10 kV.

### Statistical Analysis

Statistical analyses were performed using SPSS software. Values are reported as mean ± SD. Differences were evaluated by *t* test. Pearson correlation coefficients were used to determine the significance of linear relationships between the fatty streak lesions and plasma parameters. $P < 0.05$ was accepted as statistically significant.

### RESULTS

### Body Weight

During the 6 months of this study, the control group rabbits were healthy as demonstrated by their coat conditions and body weight gain, whereas rabbits from the high-fat/high-sucrose group showed unhealthy coat conditions and gained almost no body weight. One rabbit from the control group and 3 from the high-fat/high-sucrose group died from diarrhea during the experiment. Initial body weights for the control and

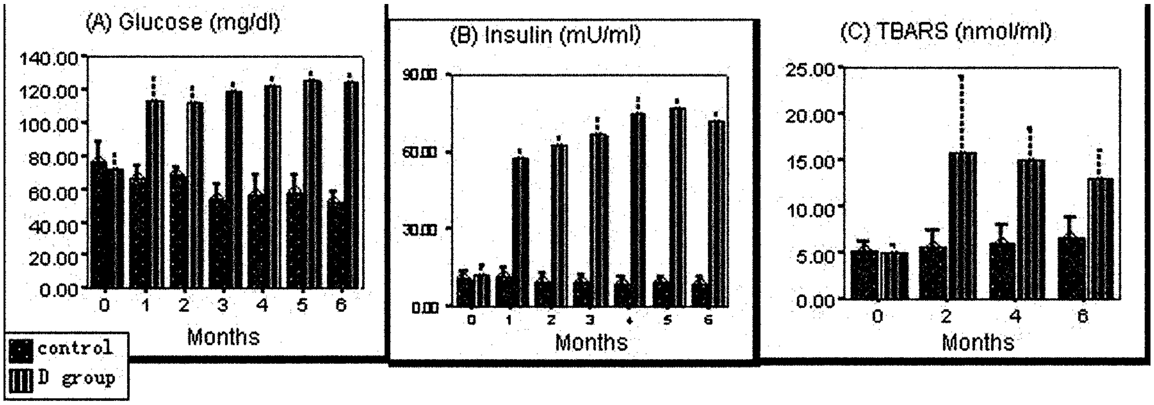

**FIGURE 1**

Plasma levels of glucose (*A*), insulin (*B*), and peroxides (TBARS, expressed as equivalents nmol malondialdehyde, MDA) (*C*) during the 6-month experimental period. The mean ± SD of 7 to 9 determinations for each experimental time point is shown. The concentrations of plasma glucose, insulin, and peroxides between the two groups were all statistically different, $P < 0.001$.

the high-fat/high-sucrose group rabbits were $2.26 \pm 0.18$ and $2.22 \pm 0.26$ kg (mean ± SD), respectively. Final body weights of the animals were $3.06 \pm 0.26$ and $2.25 \pm 0.24$ kg ($P = 0.002$, high-fat/high-sucrose group versus control group). The high-fat/high-sucrose diet was unpalatable; this might account for less weight gain of the animals fed this diet.

### Plasma Parameters

Fasting glucose concentration in plasma increased with time during feeding of the high-fat/high-sucrose diet, reaching at 5 months maximal values of $125 \pm 6$ mg/dl in the high-fat/high-sucrose group rabbits, which was 2-fold above normal control values ($57 \pm 11$ mg/dl) (Figure 1*A*). Nine animals from the high-fat/high-sucrose group had fasting plasma glucose >126 mg/dl (ranging from 126 to 160 mg/dl) at several time points and were considered to be mildly diabetic. Plasma insulin levels in the high-fat/high-sucrose group animals also increased significantly over the first 3 months and then remained at a plateau throughout the remaining experimental period (Figure 1*B*).

Peroxides in the high-fat/high-sucrose group animals increased significantly from baseline levels ($4.97 \pm 0.67$ nmol MDA/ml plasma) to a maximal value of $15.71 \pm 8.16$ nmol MDA/ml plasma at 2 months, then decreased slightly toward the end of the experiment (Figure 1*C*). Plasma NO levels in the high-fat/high-sucrose group rabbits were significantly lower than those in control rabbits (Figure 2*A*). Plasma NOS levels in the high-fat/high-sucrose group rabbits were also decreased, although there was no significant difference between

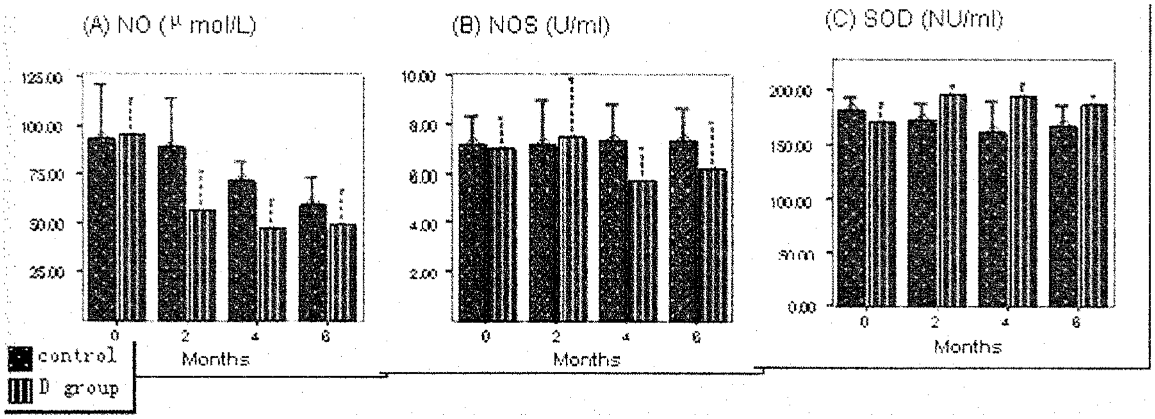

**FIGURE 2**

Plasma levels of nitric oxide (NO) (*A*), nitric oxide synthase (NOS) (*B*), and superoxide dismutase (SOD) (*C*) during the 6-month experimental period. The mean ± SD of 7 to 9 determinations for each experimental time point is shown. The levels of plasma nitric oxide and superoxide dismutase between the 2 groups were statistically different, $P \leq 0.005$. The level of nitric oxide synthase in rabbits in *D* (high-fat/high-sucrosefed group) was also decreased, but there was no significant difference between the 2 groups.

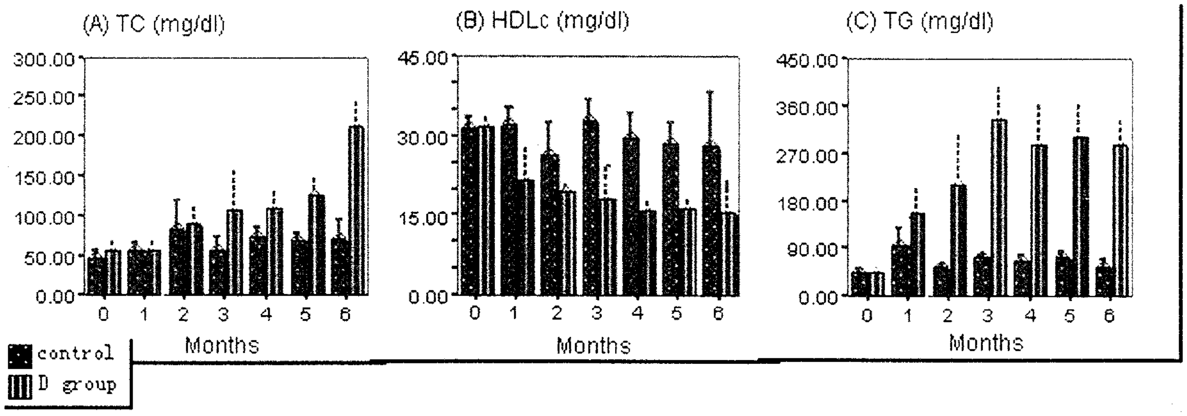

**FIGURE 3**

Plasma levels of total cholesterol (TC) (*A*), HDL cholesterol (HDLc) (*B*), and triglyceride (TG) (*C*) during the 6-month experimental period. The mean ± SD of 7 to 9 determinations for each time point is shown. The concentrations of plasma total cholesterol, HDL cholesterol, and triglyceride between the 2 groups were all statistically different, $P \leq 0.001$.

the two groups (Figure 2*B*). Plasma SOD levels increased significantly ($P = .02$) in the the high-fat/high-sucrose group animals (Figure 2*C*).

Rabbits from the high-fat/high-sucrose group showed an increasing total plasma cholesterol throughout the experimental period (Figure 3*A*). At 6 months, the cholesterol levels were the highest (212 ± 40 mg/dl, ∼3 times normal value). HDLc decreased significantly during feeding of the high-fat/high-sucrose diet (Figure 3*B*). Plasma triglycerides increased from 43 ± 9 mg/dl to a mean of 157 mg/dl (ranging from 80 to 246 mg/dl) after 1 month of high-fat/high-sucrose feeding, reaching maximal values of 333 ± 61 mg/dl (∼6 times normal value) at 3 months; similar values were found at later time points (Figure 3*C*).

### Pathological Changes in Aortas

The abdominal portions of the aortas were prone to develop fatty streak lesions in the high-fat/high-sucrose group. Relative aortic fatty streak lesion area (percent of whole area) measured by a dot-counting method were 8.58% ± 1.35% and 0.08% ± 0.06% for the high-fat/high-sucrose group and the control group, respectively ($P < 0.001$; Figure 4). Every high-fat/high-sucrose–fed rabbit developed distinct fatty streak lesions.

Inspection under TEM of cross sections of the thoracic aorta from animals fed diabetogenic diet revealed marked alterations in the pattern and integrity of the elastic laminae. Some smooth muscle cells showed altered morphology, were devoid of cytoplasmic filaments, and contained myelin figures, indicative of cellular degeneration (not shown). Macrophage-derived foam cells and small clusters of smooth muscle cells, ranging from a few to diffuse collections of several cells, were present within the subintima below the basement membrane and above the superficial elastic lamina. Most of the smooth muscle cells present within the intima were mildly activated, as judged by a slight increase in their rough endoplasmic reticulum.

On SEM, aortic arches from the high-fat/high-sucrose–fed group contained focal areas, where the normal endothelial cell pattern was absent (Figures 5*A*, *B*). The surface was rough and irregular with fibrin deposition and attached blood cells (Figure 5*B*). Sections of the aortic arch in none of the control rabbits showed abnormalities in the arrangements and integrity of the endothelial cells (Figures 5*C*, *D*).

### Correlations Between Aortic Lesion Areas and Plasma Parameters

Correlations between lesion size and plasma glucose, total cholesterol, triglyceride, insulin, and MDA levels were

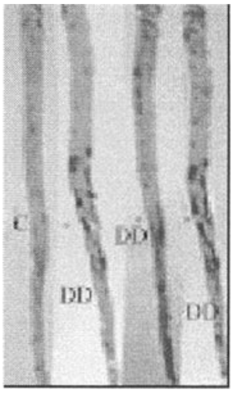

**FIGURE 4**

Fatty streak lesions in the abdominal portion of the aorta of NZW rabbits. C, control; DD, the high-fat/high-sucrose group (macrography; Sudan IV-stain).

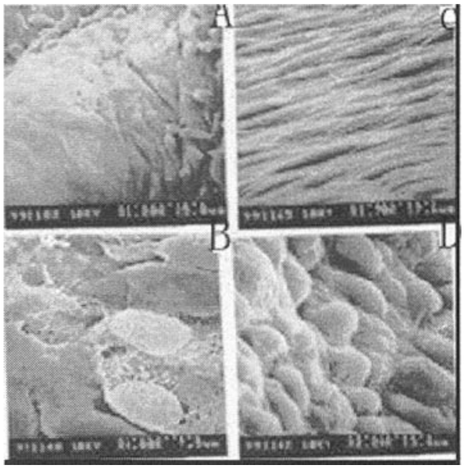

**FIGURE 5**

Scanning electron micrographs of the aortic arch of NZW rabbits. *A*, ×1200; *B*,×1700; *C*, ×1200; *D*, ×2200. (For explanations, see Results.)

significant for high-fat/high-sucrose–fed animals. Pearson's correlation coefficients are shown in Table 2.

### DISCUSSION

The aim of this study was to explore whether a diet used to induce diabetes provokes the development of aortic fatty streak lesions in New Zealand White rabbits. Our results showed that feeding a high-fat/high-sucrose diet to New Zealand White rabbits for 6 months induced significant fatty streak formation in the abdominal portions of the aortas. Structurally, abnormalities in the arrangements and integrity of the endothelial cells and marked alterations in the pattern and integrity of the elastic lamina were found; small clusters of smooth muscle cells, ranging from a few to diffuse collections of several cells, were present within the subintima. This is a novel finding, since Anitschkow demonstrated in 1913 that it was cholesterol only that caused atherosclerotic changes in the rabbit arterial intima [7]. Since that time, it had been believed that cholesterol was a necessary dietary component for inducing atherosclerosis. As far as we know, this is the first report that demonstrates that New Zealand White rabbits can develop obvious aortic fatty streaks by feeding a diabetogenic diet without added dietary cholesterol.

The mechanisms by which a high-fat/high-sucrose diet induces aortic lesions in New Zealand White rabbits needs to be further elucidated. The diabetogenic diet used in the current study has frequently been used to induce hyperglycemia and hyperinsulinemia [6, 8], but its atherogenicity has not been studied. As shown by Pearson's correlation coefficients, in rabbits of the current study, plasma glucose, TC, TG, insulin, and MDA levels all influenced fatty streak formation significantly ($P < 0.05$). Of these, plasma glucose was the parameter that showed the best correlation with fatty streaks ($r = .9$, $P < 0.001$). Because plasma glucose and triglyceride levels were elevated at early time points and remained high during the experimental period in diabetogenic diet fed rabbits of this study, these 2 parameters may be the main factors contributing to fatty streak formation. Kunjathoor and colleagues [9] demonstrated that streptozotocin-induced hyperglycemia was a prime contributor to accelerated fatty streak formation in BALB/c mice under conditions of high plasma lipid levels. In the current study, the high-fat/high-sucrose diet induced hyperglycemia, hyperinsulinemia, oxidative stress, and atherogenic lipid profiles in New Zealand White rabbits. Therefore, with respect to the atherogenicity of this diet, hyperglycemia should be considered as a prime cause. Under the condition of hyperglycemia, lipoproteins may be glycated, making them more readily taken up by scavenger receptors on cells of the artery wall; glycation of matrix molecules occurs, which could provide a stronger trapping network for lipoproteins penetrating vascular spaces [10]. In recent years, much attention has been given to oxidative stress as a potentially important factor in the pathogenesis of many diseases, including atherosclerosis, cancer, and diabetes [11]. A role for oxidized low-density lipoprotein (OxLDL) lipids in the development of atherosclerotic lesions has also been firmly established over the past several years [12]. In the present study, oxidative stress induced by the high-fat/high-sucrose diet, as indicated by significantly increased plasma peroxides in the high-fat/high-sucrose group, should enhance the oxidation of LDL. The oxidation of LDL confers many biological properties on the molecules that render the lipoproteins more atherogenic; for example, OxLDL is avidly scavenged by macrophages and leads to macrophage-derived foam cell formation [12]. In this current

**TABLE 2**
Correlations between aortic lesion areas and the main plasma parameters

| | | TC | TG | HDLc | Glucose | Insulin | MDA |
|---|---|---|---|---|---|---|---|
| Lesion | Pearson correlation | .866** | .863** | −.465 | .900** | .248 | .775* |
| | Significance (2-tailed) | .003 | .003 | .208 | .001 | .521 | .014 |

*Correlation is significant at the .05 level (2-tailed).
**Correlation is significant at the .01 level (2-tailed).

study, macrophage-derived foam cells within the subintima of aortic samples from high-fat/high-sucrose diet–fed rabbits were observed. In addition, a direct role of hyperglycemia and hypertriglyceridemia on blood viscosity needs to be taken into account. The hemorheologic-hemodynamic theory [13] suggests that increased blood viscosity may accelerate atherogenesis. Increased blood viscosity is found in association with many major risk factors for accelerated atherogenesis, including hypertension, cigarette smoking, diabetes mellitus, obesity, and hyperfibrinogenemia [13].

Increased atherosclerosis is a common characteristic in diabetic patients [1, 2]. Studies of the pathogenic components and of the mechanisms of accelerated atherosclerosis in diabetes are hindered by inadequate animal models [4–6, 9]. Diabetic animal models may be created by injections of streptozotosin (STZ) or alloxan [4, 5, 9], or feeding diabetogenic diets to animals [6]. On the basis of the STZ- or alloxan-induced insulin-dependent type 1 model of diabetes mellitus, cholesterol has been added to the diet to produce accelerated atherosclerosis in diabetic animals [4, 5, 9]. In the present study, however, significant fatty streak formation in New Zealand White rabbits was induced solely by high-fat/high-sucrose diet, without adding dietary cholesterol. Schreyer and colleagues first reported that 40% of C57BL/6 mice fed a high-fat/high-sucrose diet exhibited small fatty streak lesions [6]. In comparison, in our study, 100% of rabbits developed distinct fatty streak lesions.

In summary, our results suggest that a diabetogenic diet may induce atherosclerosis in rabbits by altering lipid and glucose metabolism, as well as producing oxidative stress. Therefore, New Zealand White rabbits fed a high-fat/high-sucrose diet provide a convenient model in which to identify dietary and diabetogenic factors contributing to accelerated atherosclerosis, and to study the mechanisms underlying these effects.

### REFERENCES

[1] Wood, D. (1998) European and American recommendations for coronary heart disease prevention. *Eur. Heart. J.,* **19,** A12–A19.

[2] Semenkovich, C. F., and Heinecke, J. W. (1997) The mystery of diabetes and atherosclerosis—time for a new plot. *Diabetes,* **46,** 327–334.

[3] Watts, G. F., and Playford, D. A. (1998) Dyslipoproteinaemia and hyperoxidative stress in the pathogenesis of endothelial dysfunction in non-insulin dependent diabetes mellitus: An hypothesis. *Atherosclerosis,* **141,** 17–30.

[4] Dixon, J. L., Stoops, J. D., Parker, J. L., Laughlin, M. H., Weisman, G. A., and Sturek, M. (1999) Dyslipidemia and vascular dysfunction in diabetic pigs fed an atherogenic diet. *Arterioscler. Thromb. Vasc. Biol.,* **19,** 2981–2992.

[5] Simionescu, M., Popov, D., Sima, A, Hasu, M., Costache, G., Faitar, S., Vulpanovici, A., Stancu, C., Stern, D., and Simionescu, N. (1996) Pathobiochemistry of combined diabetes and atherosclerosis studied on a novel animal model. The hyperlipemic-hyperglycemic hamster. *Am. J. Pathol.,* **148,** 997–1014.

[6] Schreyer, S. A., Wilson, D. L., and LeBoeuf, R. C. (1998) C57BL/6 mice fed high fat diets as models for diabetes-accelerated atherosclerosis. *Atherosclerosis,* **136,** 17–24.

[7] Finking, G., and Hanke, H. (1997) Nikolaj Nikolajewitsch Anitschkow (1885–1964) established the cholesterol-fed rabbit as a model for atherosclerosis research. *Atherosclerosis,* **135,** 1–7.

[8] Surwit, R. S., Kuhn, C. M., Cochrane, C., et al. (1998) Diet-induced type II diabetes in C57BL/6J mice. *Diabetes,* **37,** 1163–1167.

[9] Kunjathoor, V. V., Wilson, D. L., and LeBoeuf, R. C. (1996) Increased atherosclerosis in streptozotocin-induced diabetic mice. *J. Clin. Invest.,* **97,** 1767–1773.

[10] Lyons, T. J. (1992) Lipoprotein glycation and its metabolic complications. *Diabetes,* **41**(Suppl. 2), 67–73.

[11] Mullarkey, C. J., Edelstein, D., and Brownlee, M. (1990) Free radical generation by early glycation products: A mechanism for accelerated atherogenesis in diabetes. *Biochem. Biophys. Res. Commun.,* **173,** 932–939.

[12] Hunt, J. V., Bottoms, M. A., Clare, K., Skamarauskas, J. T., and Mitchinson, M. J. (1994) Glucose oxidation and low-density lipoprotein-induced macrophage ceroid accumulation: Possible implications for diabetic atherosclerosis. *Biochem. J.,* **300,** 243–249.

[13] Sloop, D. (1999) Viewpoint: A critical analysis of the role of cholesterol in atherogenesis. *Atherosclerosis,* **142,** 265–268.

